# The potential of training specialist oncology nurses in real-life reporting of adverse drug reactions

**DOI:** 10.1007/s00228-021-03138-5

**Published:** 2021-05-12

**Authors:** M. Reumerman, J. Tichelaar, R. van Eekeren, E. P. van Puijenbroek, M. C. Richir, M. A. van Agtmael

**Affiliations:** 1 Department of Internal Medicine, AmsterdamUMC, Location VUmc, De Boelelaan 1117, Amsterdam, 1081 HV The Netherlands; 2 Research and Expertise Center in Pharmacotherapy Education (RECIPE), De Boelelaan 1117, Amsterdam, 1081 HV The Netherlands; 3 The Netherlands Pharmacovigilance Centre Lareb, Hertogenbosch, The Netherlands; 4 Groningen Research Institute of Pharmacy, PharmacoTherapy, - Epidemiology & -Economics, University of Groningen, Groningen, The Netherlands

**Keywords:** Oncology, Medical education, Adverse drug reports, Pharmacovigilance, Specialist oncology nurses, Clinical effects, Long term effects

## Abstract

Specialist oncology nurses (SONs) have the potential to play a major role in monitoring and reporting adverse drug reactions (ADRs); and reduce the level of underreporting by current healthcare professionals. The aim of this study was to investigate the long term clinical and educational effects of real-life pharmacovigilance education intervention for SONs on ADR reporting. This prospective cohort study, with a 2-year follow-up, was carried out in the three postgraduate schools in the Netherlands. In one of the schools, the prescribing qualification course was expanded to include a lecture on pharmacovigilance, an ADR reporting assignment, and group discussion of self-reported ADRs (intervention). The clinical value of the intervention was assessed by analyzing the quantity and quality of ADR-reports sent to the Netherlands Pharmacovigilance Center Lareb, up to 2 years after the course and by evaluating the competences regarding pharmacovigilance of SONs annually. Eighty-eight SONs (78% of all SONs with a prescribing qualification in the Netherlands) were included. During the study, 82 ADRs were reported by the intervention group and 0 by the control group. This made the intervention group 105 times more likely to report an ADR after the course than an average nurse in the Netherlands. This is the first study to show a significant and relevant increase in the number of well-documented ADR reports after a single educational intervention. The real-life pharmacovigilance educational intervention also resulted in a long-term increase in pharmacovigilance competence. We recommend implementing real-life, context- and problem-based pharmacovigilance learning assignments in all healthcare curricula.

**Supplementary information:**

The online version contains supplementary material available at 10.1007/s00228-021-03138-5.

## Introduction

Pharmacovigilance centers have a major role in the post-marketing monitoring of drug safety for which the spontaneous reporting system is the best recognized. [1, 2] The main function of this system is the early detection of new, rare, or serious adverse drug reactions (ADRs) or those with a long time of onset, which may not have been detected in pre-marketing trials or post-marketing surveillance studies. [3] The major limitation of this system is the high level of underreporting of ADRs, with an estimated median under-reporting rate across 37 studies of 94% (interquartile range 82–98%). [4, 5] This underreporting may hinder optimal ADR monitoring, may mask true side effect profiles, and extend exposure to possibly harmful drugs. [4, 6].

Despite the diverse and numerous interventions to decrease the level of underreporting by physicians and pharmacists, they have not been as successful as anticipated. [7] Patient reporting, especially in some European countries, has increased the number of ADR-reports (doubled the number in the Netherlands), has provided an additional source of information and has strengthened medication safety signals. [8, 9] The downside is that patient reports contain more subjective elements of information and less clinically related information. [10, 11] The involvement of specialist nurses may increase the number of high quality reports, because (specialist) nurses administer most drugs, they have prescribing privileges (specialist nurses), they are often aware of the occurrence of ADRs in their patients and they are an objective source of information. [5, 12–14].

Many studies have already demonstrated the importance of specialist nurses to pharmacovigilance. [13–15] This is especially true for medical specialties that use high risk medications and which have low reporting rates, such as hematology and oncology. [16] Our previous study involving specialist oncology nurses (SONs) also showed that, after an educational intervention, these specialist nurses were adequately prepared, had sufficient knowledge and adequate abilities, and had a positive attitude to monitoring and reporting ADRs. [17].

Although specialist nurses seem prepared for their role in pharmacovigilance, the rate of underreporting by nurses in general is especially high. For instance, in the Netherlands the reporting rate per 1000 nurse-years is 0.80. [18, 19] Multiple studies have investigated the reasons and determinants of underreporting [4, 5], which can be broadly classified in three groups: (i) ‘this is not part of my profession’ i.e. attitude about professional activities (financial incentives, legal aspects, and ambition to publish); (ii) ‘this is not part of my knowledge domain’ i.e. ADR-related knowledge and attitudes (complacency, insecurity, diffidence, indifference, and ignorance); and (iii) ‘this is not part of my interest’ i.e. excuses made by professionals (lethargy). Despite this knowledge, educational interventions for nurses in general have not been specifically designed to address these determinants.

A weak point of previous educational interventions is the use of outdated educational techniques and surrogate outcome measures (attitude and knowledge) to predict the effectiveness of an intervention. Our recent literature review showed an urgent need for more innovative learning initiatives in pharmacovigilance training [20] and a recent study stressed the use of clinical and real-life continuing learning initiatives. [21] It also advised studying the long-term effects and clinical value of educational initiatives. [20].

We chose to study SONs because they support current physicians and pharmacists and high risk medications are used in oncology and ADR reporting rates are low. [22, 23] Therefore, the aim of this study was to investigate the long-term clinical and educational effects of a real-life pharmacovigilance educational intervention for healthcare professionals, by analyzing the quantity and quality of their ADR reports and assessing their attitudes, skills, and knowledge of pharmacovigilance.

## Methods

This longitudinal prospective cohort study to evaluate the long-term effects of real-life pharmacovigilance education was carried out in the three colleges that offer a course on prescribing for SONs in the Netherlands.

### Setting

**Fig. 1:**
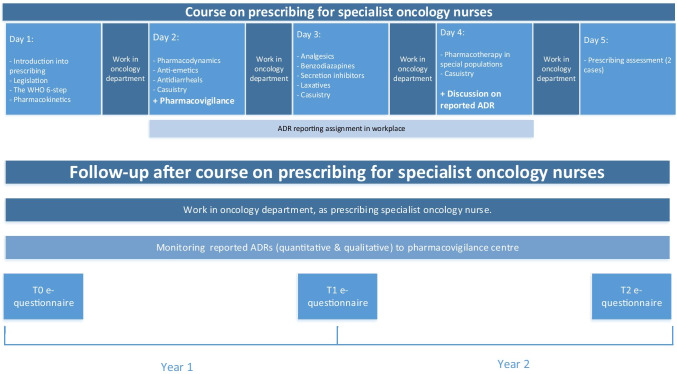
Overview of the course curriculum and follow-up period

Three colleges (“The Amstel Academy”, “Radboudumc Health Academy”, and “Wenckebach Instituut UMCG”) offer registered SONs a nationally established course to enable them to qualify to prescribe a limited set of frequently prescribed drugs (anti-diarrhea drugs, anti-emetics, non-opioid analgesics, and benzodiazepines). SONs follow the course in addition to their work in different (mostly non-academic) hospitals in the Netherlands. The course consists of 4 days (6 h/day) of lectures and small group exercises spread over half a year, and is completed by a prescribing assessment. The national course overview is displayed in Fig. 1a.

The Amstel Academy, the college with the largest group size is the only college that also provided the pharmacovigilance intervention. The intervention consisted of a lecture on basic pharmacovigilance, a real-life reporting assignment of (unknown, exceptional, or unexpected) ADRs during work, and group discussion of the reported ADRs. The course was led by a pharmacotherapy teacher (MR & TS) and assessor from the Pharmacovigilance Center Lareb (R.v.E.). After the prescribing assignment, participants evaluated their experience by writing a short portfolio essay on their ADR report.

### Population and inclusion

All SONs enrolled in the courses in the three colleges in the Netherlands from November 2015 – September 2017 were invited to participate in this study voluntarily. Participation included giving written or digital approval for an assessor from the Pharmacovigilance Centre Lareb (RvE) to search their database for submitted ADR reports (using email-address, name and institution). Additionally, SONs were asked to fill in an e-questionnaire at three different times after the course, to analyze changes in attitudes and knowledge after the prescribing qualification course. Inclusion was done by personal invitation by the local course coordinator, via e-mail.

### Instruments

Three aspects of ADR reporting were evaluated: the number of reports submitted before and after the intervention; the quality and relevance of these reports; and the competence of SONs with regard to pharmacovigilance and ADR reporting (Fig. 1b).

The number of ADR reports submitted was measured by extracting all reports submitted by the participants from the Lareb database up to 2 years after course completion. This was done by drafting a query based on their email address and hospital name, from 2015 to March 2019. All retrieved reports were checked manually.

The ClinDoc algorithm [24] was used to determine the quality of the clinical documentation of each ADR report. Two researchers (MR and RVE) individually assessed the first five reports, to check whether they scored reports in a similar way (all reports had the same score). As this was the case, the other reports were divided among the two researchers. Two reports were difficult to score and were discussed before agreement was reached on the score. Aspects that indicate relevance of the ADR report were scored automatically (seriousness) or manually, using the report form or pharmacovigilance center assessment information (labeling information, off-label use, additional monitoring and withdrawal of chemotherapeutic treatment).

The competence of the SONs was evaluated with three e-questionnaires (Supplementary Table 1), using Castor EDC. The e-questionnaires consisted of three parts – baseline characteristics, intention/attitudes, and knowledge/skills of the SONs (16questions). The first e-questionnaire was sent within 2 weeks after the prescribing assessment and the second and third at 1-year intervals. If participants did not respond, two reminders were sent at a 2-weekly intervals.

**Table 1:** Baseline characteristics of specialist oncology nurses

SONs characteristics			
	Intervention group	Control group	Significance
Total number of SONs following a prescribing course	70	43	-
Total number of SONs in study (% of total)^a^	65/70 (93%)	23/43 (53%)	< 0.001
2015 (% of SONs in course)	28/32 (88%)	12/22 (55%)	-
2016 (% of SONs in course)	29/30 (97%)	11/21 (52%)	
2017 (% of SONs in course)	8/8 (100%)	-	
Participant characteristics			
Sex, female (%)^b^	54/65 (83%)	23/23 (100%)	0.035
Age (yrs), median age group (IQR)^b^	45–55 (35–45 / 45–55)	35–45 (35–45 / 45–55)	0.126
Experience (yrs), median (IQR) ^a^	15 (9–23)	13 (7–15.5)	0.089
Daily prescriptions, median (IQR) ^a^	1 (0–2)	0 (0–1)	0.101
Previous training			
Education on adverse drug reactions (%)^a^	37 (57%)	12 (55%)	0.694
Education on reporting adverse drug reactions (%)^a^	5 (8%)	2 (9%)	0.879
Previous report ^a^	5 (8%)	1 (4%)	0.584
Work environment			
Non-academic hospital ^a^	59 (91%)	20 (87%)	0.604
Outpatient clinic ^a^	29 (45%)	14 (61%)	0.180
Oncology/hematology department ^a^	42 (65%)	18 (78%)	0.227
Number of different hospitals/clinics (median number of SONs in each hospital)	18 (3.3)	10 (2.2)	-

^a^Mann-Whitney U test (alpha 0.5 and *p* < 0.05)

^b^Chi-squared (alpha 0.5 and *p* < 0.05)

### Data analysis

All data were imported in SPSS Statistics 22 (IBM Corp.; Armonk, New York). Descriptive statistics were used to report frequencies and means/median/standard deviations (SD) and interquartile range (IQR) of survey results. Differences between baseline characteristics were compared using Chi-squared and Mann–Whitney U test (alpha 0.5 and *p* < 0.05). Attitude and knowledge scores between intervention and control groups of SONs were computed with.

Mann–Whitney U test, with Bonferroni’s correction (*p* < 0.0025). Reported ADRs were categorized in two groups: 1. Reports submitted during the intervention and 2. Reports submitted after the educational intervention. The prescribing assessment was used as cut-off date. Reporting rates per 1000 nurse years in the study (intervention group) and the national average were compared by dividing the ADR-reporting rates (according to the Pharmacovigilance Center Lareb) [18, 19] by the number of BIG-registered nurses (including specialist nurses) [18]. Open questions were analyzed using thematic analysis [25].

### Ethical aspects

This study did not fall under the scope of the Dutch Medical Research Involving Human Subjects Act. (reference number 2017.034). Participation was voluntary and based on informed consent. Participants did not receive credit or other incentives to participate. The ethics review board of the Netherlands Association for Medical Education (NVMO) reviewed the protocol and approved this study (ID: 692 & 826).

## Results

All SONs enrolled in the three prescribing qualification courses in the Netherlands (*n* = 113) were invited to participate in this study, 88 (78%) of whom signed the informed consent form (65 in the intervention group and 23 in the control group). All included SONs filled in the first questionnaire (T0), 73 (83%) filled in the second questionnaire (T1), and 62 (70%) filled in all three questionnaires (T2).

At baseline, no significant differences were found in the characteristics of the SONs in the intervention and control groups, except for a sex difference (more female SONs in the control group). Overall, 76 SONs were women, the median age was 45–55 years, and SONs had a median of 14 years of clinical experience as a nurse. Most SONs worked on an oncology and hematology ward (68%) in a non-academic hospital (90%). While 49 SONs (56%) reported having had a lecture on ADRs before the prescribing qualification course, only 7 SONs (8%) indicated that their previous training covered when or how to report an ADR. Before starting the course, 6 SONs (5%) had reported one or more ADRs to the Netherlands Pharmacovigilance Centre Lareb (Table 1).

### Clinical outcomes

#### ADR reporting (number)

During the study period, 82 ADR reports (concerning 220 different ADRs) were reported to Lareb. All intervention-group SONs submitted at least one ADR report during and after the course, whereas none of the control-group SONs did. Five participants submitted more than one ADR report during the educational intervention. Seven SONs (11%) submitted one or more ADR reports after the intervention, accounting for 11 additional reports (Table 2a).

**Table 2:** ADR reports characteristics

A. ADR report characteristics								
				Intervention group		Control group		P-value
Participants with ≥ 1 ADR report (% of total)^a^				65 (100%)		0 (0%)		< 0.001
Total number of ADR reports (average for each SON)^b^				82 (1.32)		0 (0)		< 0.001
Total number of reported ADRs (average for each SON) ^b^				220 (3.55)		0 (0)		< 0.001
Participants with > 1 report (% total)^c^				12 (18%)		0 (0%)		0.032
Participants with > 1 report during educational intervention (% of total)				5 (8%)		-		na
Number of extra reports during educational intervention (average for each SON)				6 (0.1)		-		
Participants with ≥ 1 report after course (% of total)^c^				7 (11%)		0		0.183
Number of additional reports after course (average for each SON)^b^				11 (0.18)		0		0.049
Number of additional reports in the first year after the course^b^				7 (11%)		0		0.106
Number of additional reports in the second year after the course^b^				4 (6%)		0		0.230

B. ClinDoc scores of ADR reports								
	n		Well documented		Moderately documented		Poorly documented	
During prescribing qualification course	71		60 (85%)		11 (15%)		0	
After prescribing qualification course	11		9 (82%)		2 (18%)		0	
Total	82		69 (84%)		13 (16%)		0	

C. Characteristics of reported ADRs								
					n		%	
Total number of ADR reports					82			
Non-serious reports					47		57%	
Serious reports					35		43%	
Off-label use					3		4%	
Drug under additional monitoring					14		17%	
ADR cause of withdrawal of chemotherapeutic drug					14		17%	
Total number of drugs involved in ADRs					220			
Chemotherapeutics					150		68%	
Monoclonal antibodies		ATC-L01XC			37		17%	
Pyrimidine analogues		ATC-L01BC			37		17%	
Platinum compounds		ATC-L01XA			31		14%	
Taxanes		ATC-L01CD			15		7%	
Mammalian target of rapamycin kinase inhibitors		ATC-L01EG			5		2%	
Anti-androgens		ATC-L02BB			4		2%	
Other chemotherapeutics		-			21		10%	
Concomitant drugs with chemotherapy					21		10%	
Diagnostic agents					9		4%	
Other drugs (not chemotherapy related)					40		18%	
Total number ADRs classified by System Organ Class (MedDRA-classification)					220			
Skin and subcutaneous tissue disorders					41		19%	
Gastrointestinal disorders					34		15%	
Nervous system disorders					30		14%	
General disorders and administration site conditions					21		10%	
Respiratory, thoracic and mediastinal disorders					14		6%	
Eye disorders					10		5%	
Vascular disorders					7		3%	
Psychiatric disorders					7		3%	
Infections and infestations					7		3%	
Cardiac disorders					7		3%	
Other					41		19%	
Labeling information in 4.8 ADR section of SmPC								
Labelled					154		70%	
Not labelled					66		30%	

One report may contain more than one ADR. Seriousness can be coded for each ADR or for the ADR report; we chose to mention seriousness for a report, since this considers one patient

^a^chi-squared without Fisher’s exact test

^b^Mann-Whitney U test

^c^chi-squared with Fisher’s exact test B: ClinDoc scores of the reported ADRs during and after the intervention (Since only specialist oncology nurses in the intervention group reported ADRs, the ClinDoc scores are only shown for this group). C: Characteristics of adverse drug reactions (ADRs) reported in this study. Drugs were classified according to the ATC-classification, ADRs were classified according to System Organ Class by MedDRA

#### ADR reporting (quality)

Since only the intervention-group reported ADRs, the reporting quality of the intervention and control groups could not be compared. ClinDoc scores higher than 75% indicate a well-documented ADR report; 69 of the 82 (84%) ADR reports had a score higher than 75% (Table 2b / Supplementary Fig. 1). The scores for the reports submitted during and after the intervention were not significantly different (p = 0.227).

#### ADR characteristics

Most of the ADR reports concerned monoclonal antibodies (17%), pyrimidine analogues (17%) and platinum compounds (14%) and associated skin reactions (19%), gastrointestinal disorders (15%), and nervous system disorders (14%) (Table 2c). Thirty-five (43%) reports met criteria for seriousness (CIOMS), with hospitalization being mentioned in 24 reports (Table 2c). The main serious reactions reported were peripheral edema, dizziness, diarrhea, and dehydration. More serious conditions were acute myocardial infarction (*n* = 2), pneumonitis (*n* = 2), toxic skin eruption (possible Stevens-Johnson syndrome) (*n* = 1), anaphylactic reaction (*n* = 2). Four fatal reactions occurred and involved ascites, cardiogenic shock, hypovolemia due to diarrhea and gastrointestinal hemorrhage, and pulmonary embolism. Although most (70%) of the reported reactions were mentioned in 4.8 section (Side effects) of the Summary of Product Characteristics (SmPC) 30% of the reported reactions were unexpected.

### Educational outcomes

#### Intentions and attitudes

The intervention-group SONs had significantly higher scores for intention to report unknown and serious ADRs than the control group SONs up to 2 years after the prescribing qualification (Table 3). All SONs thought that reporting ADRs would “contribute to medication safety”, “improve patient safety”, and would “educate others about drug risks”; however, the intervention-group had a more positive attitude to these outcomes than the control group (*p* < 0.05). The intervention-group SONs were significantly more likely to consider that reporting “disrupts the normal workflow” and “is time consuming” at all-time points (*p* < 0.05). All SONs agreed that physicians and pharmacists are important healthcare professionals with regard to reporting ADRs, but only the intervention-group SONs considered themselves to be an important healthcare professional for reporting ADRs (*p* < 0.05).

**Table 3:** Intention, attitudes, and opinions regarding the reporting of adverse drug reactions

	Intervention group			Control group			Intervention vs control group		
	T1 (*n* = 65)	T2 (*n* = 54)	T3 (*n* = 47)	T1 (*n* = 23)	T2 (*n* = 19)	T3 (*n* = 15)	T1 vs T1	T2 vs T2	T3 vs T3
A. Could you indicate how likely it is you will report an ADR to Lareb in the following situations:									
I intend to report all ADRs that I encounter to the competent authority	4.967 (1.5941)	4.037 (1.1321)	4.064 (1.5095)	2.773 (0.9223)	2.526 (1.1723)	2.800 (1.1464)	< 0.001	< 0.001	0.004
I intend to report unknown ADRs that I encounter to the competent authority	5.633 (1.3400)	5.574 (1.2378)	5.915 (1.1947)	3.091 (1.5708)	2.737 (1.5931)	3.533 (0.5164)	< 0.001	< 0.001	< 0.001
I intend to report serious ADRs that I encounter to the competent authority	6.033 (1.3144)	5.741 (1.1358)	5.660 (1.2731)	3.500 (1.6833)	3.579 (1.7100)	3.000 (1.1952)	< 0.001	< 0.001	< 0.001

B. How likely do you think the following outcomes will be if you report a serious ADR:									
Contributes to the safe use of medicines	6.450	6.519	6.298	5.091	5.263	5.067	< 0.001	< 0.001	< 0.001
Improves patient safety	6.433	6.370	6.149	5.364	5.842	5.267	< 0.001	0.028	0.001
Educates others about drug risks	5.983	5.963	5.702	4.045	3.947	3.733	< 0.001	< 0.001	< 0.001
Personally beneficial	5.467	5.500	4.979	3.455	3.000	2.667	< 0.001	< 0.001	< 0.001
Time consuming to report	4.350	4.537	4.940	3.864	3.526	3.933	0.205	0.002	< 0.001
Disrupts the normal workflow	4.233	4.796	4.255	3.364	3.368	3.400	0.025	< 0.001	0.045
Increases risk of malpractice	2.217	1.870	1.745	2.955	2.421	2.933	0.020	0.097	0.001
Breaks trust with patients	1.750	1.722	1.745	2.545	1.947	2.200	0.002	0.450	0.102

C. How important are these healthcare professionals in reporting ADRs									
Physician	4.250	4.278	4.255	3.864	3.421	3.867	0.173	0.001	0.269
Pharmacist	4.233	4.407	4.298	4.000	4.421	4.200	0.402	0.958	0.768
Specialist oncology nurses	4.283	4.370	4.298	1.955	2.263	2.000	< 0.001	< 0.001	< 0.001

D. Opinion regarding (current) education in pharmacovigilance:									
Pharmacovigilance should be included as a core topic in the curriculum of all prescribers	4.483	4.574	4.574	3.273	3.316	3.533	< 0.001	< 0.001	< 0.001
Pharmacovigilance is well covered (up to now) in my curriculum	3.867	4.296	4.085	2.364	2.579	2.533	< 0.001	< 0.001	< 0.001
With my knowledge I am well prepared for my role in pharmacovigilance	4.333	4.426	4.404	2.636	2.737	2.667	< 0.001	< 0.001	< 0.001
I do not know how I should report an ADR to the relevant authorities	1.633	1.370	1.447	3.364	3.263	3.267	< 0.001	< 0.001	< 0.001

E. Opinion regarding current and future role in pharmacovigilance:									
Students can report ADRs during their clerk/internships	4.200	4.519	4.404	2.545	2.474	2.133	< 0.001	< 0.001	< 0.001
Reporting known ADRs makes no significant contribution to the reporting system	1.950	2.593	3.064	3.227	3.158	3.333	< 0.001	0.044	0.351

(ADR) and pharmacovigilance (education) of specialist oncology nurses at T0 (direct after prescribing course), T1 &T2 (1 and 2 year after the course). A & B (on a 7-point Likert scale) C-E (on a 5-point Likert scale). Mann–Whitney U test, with Bonferroni’s correction (alfa < 0.0025) was used

#### Knowledge (quantitative)

The intervention-group SONs had significantly (*p* < 0.05) better scores on most (8/11) dichotomous knowledge questions directly after course completion compared with the control group. Three questions regarding “anonymous reporting”, “adverse experiences with cosmetics”, and “natural and homeopathic products” were answered correctly by only 50% of the SONs in both groups. After 2 years, the intervention group still significantly outperformed the control group on most questions (6/11) (Supplementary Table 2).

#### Skills (qualitative)

Analysis of the open-ended questions showed that the intervention-group SONs were more aware of “what to do when they suspect an ADR”. They would significantly more often perform “causality assessments”, would try to reduce symptoms by “stopping the suspected drug”, “search for alternative drug”, “lower dose”, or even perform a “re- or dechallenge”. They also spontaneously mentioned “reporting the ADR” more often than did the control-group SONs (Supplementary Fig. 2).

When asked what essential information is needed for a qualitatively good report, the intervention-group SONs reported more options. The control-group SONs primarily mentioned “description of the adverse drug reaction”, “suspected drug” and “co-medication”, whereas the intervention-group SONs also mentioned “start-stop dates”, “latency time”, “re- and dechallenge information” and would supply additional information and laboratory data (Supplementary Fig. 3).

## Discussion

SONs who received the context-based pharmacovigilance educational intervention submitted significantly more, qualitatively good ADR reports than did SONs who did not receive the intervention in the first 2 years after they completed a prescribing qualification course. The latter group did not submit any ADR reports. The SONs who received the intervention considered themselves more ready for their role in pharmacovigilance, had sustainable and more positive attitudes, and had a better knowledge of pharmacovigilance and ADR reporting up to 2 years after course completion. Additionally, their ADR reports provided valuable, relevant, and well-documented information in a specialized field with a low reporting rate.

SONs who received pharmacovigilance training had a reporting rate of 84.6 per 1000 nurse-years in the first two years after the intervention. This made the SONS 105 times more likely to report an ADR after the prescribing course than the national reporting average for nurses in the Netherlands [18, 19]. This major increase should be interpreted with caution since reporting rates among nurses in the Netherlands are lower than in other European countries and the specialist oncology nurses in our study would be expected to be more aware of ADRs and report more frequently, because they have prescribing authority.

Our results were similar to those of other educational interventions in nurses. Bäckström et al. [26] reported a large (tenfold) increase in ADR reporting rates among nurses who had followed repeated educational lectures. Other interventions for non-nurse healthcare professionals (physicians/pharmacists) had a similar effect on ADR reporting rates (increase of 2- to sixfold). [27–30] These educational interventions are typically short (1 h) lectures or workshops, with follow-up reminders, educational materials or emails, which makes them more difficult to implement.

Despite our clinically relevant increase in the number of ADR reports, we did not observe a further growth during the follow-up period, 11% of SONs submitted an ADR report in the first year after the intervention but only 6% in the second year. Previous intervention studies also reported that under-reporting increased with increasing time after an intervention [7, 25–28]

Hazell et al. [4] and Lopez-Gonzalez et al. [5] identified three distinct groups of determinants of underreporting (Supplementary Table 3). Our real-life pharmacovigilance intervention improved “attitudes relating to professional activities” and “increased ADR-related knowledge and attitudes” (the first two determinants). Our intervention did not address the third determinant: “excuses made by professionals”. These excuses include: “I don’t have time”, “I have different care priorities”, and “reporting is bureaucratized”, and are factors not easily addressed in educational interventions. Therefore, we strongly recommend the development of guidelines and national/international regulations for reporting ADRs. If medical directors or policy makers put more emphasis on the importance of reporting ADRs, healthcare professionals will eventually change their care priorities and time will be made available for reporting ADRs during work. Many initiatives, such as making reporting forms more accessible [34], reporting with apps or discharge letters and Adverse Drug Event Manager (ADEMs) [35] teams, have made ADR reporting more effective; however, these initiatives have not succeeded in changing healthcare professionals’ views on pharmacovigilance and ADR reporting.

The strength of this study lies in its design, a prospective cohort study with a 2-year follow-up for clinical and educational outcomes. Moreover, the participants formed a homogenous group as they all had a similar educational background and had followed a nationally established prescribing course. In total, 78% of the entire population of SONs in the Netherlands with a prescribing qualification were included. Lastly, the dropout rate was low. No SON withdrew their consent to search for reported ADRs in the National Pharmacovigilance Database and e-questionnaire response rates were 100% (directly after prescribing assessment), and 84% and 73% at 1 and 2 years of follow-up, respectively.

The main limitation of this study is the relatively small control group of 23 SONs. Since the included SONs were probably the ones most interested in pharmacovigilance, the results of this group could therefore be an overestimation of the practice, knowledge, and attitude to ADR reporting of SONS in general. A second limitation could be caused by the educational intervention itself. The intervention consisted of a real ADR report, analysis, class discussion and a portfolio assignment which took much longer than a normal ADR report. The extensiveness of the intervention could have influenced the reporting rates by conditioning SONs to expect ADR reporting to be time consuming. This is borne out by the finding that the intervention-group SONs had significantly higher scores on questions about the time required (“time consuming to report” and “disrupts the normal workflow”) (Table 3). A second study where SONs perform a short (and quick) ADR reporting assignment could distinguish between the two reasons.

Taking these limitations into account, this study is the first to show a significant and clinically relevant increase in the quantity and quality of ADRs reported after a single educational intervention. Although we studied SONs, we hypothesize that training other healthcare professionals will improve the level of ADR reporting. Therefore, we would recommend that other healthcare curricula (medicine, pharmacy, or nursing) incorporate this simple real-life, problem- and context-based pharmacovigilance educational intervention as it fits perfectly into the proposed curriculum. [21] Further research is needed to analyze whether a shorter version of the reporting assignment will further encourage ADR reporting. Although our findings are encouraging, reporting rates were limited by “Excuses made by professionals”, an aspect not adequately addressed in our educational intervention. This emphasizes the importance of increasing the time available in medical curricula to teach medication safety and pharmacovigilance, so as to increase medication awareness. We recommend guidelines and national/international regulations in clinical practice to maximize reporting rates.

## Supplementary Information

Below is the link to the electronic supplementary material.

Supplementary file1 (DOCX 65 KB)

## Data Availability

The data that support the findings of this study are available from the corresponding author upon reasonable request.

## Declarations

### Originality and agreement statement

All off the authors declare that this work has not been, and will not be published, in whole or part in any other journal and agree to the contents of the manuscript in its submitted form.

### Principal investigator

The authors confirm that the PI for this paper is Dr. J.Tichelaar that he had direct clinical responsibility for patients.
