# Supplementary material for: The potential of training specialist oncology nurses in real-life reporting of adverse drug reactions
Source: Eur J Clin Pharmacol. 2021 May 12;77(10):1531–42. doi: 10.1007/s00228-021-03138-5 (PMC8440292; doi:10.1007/s00228-021-03138-5)
Supplement: Supplementary file 1 — Supplementary file1 (DOCX 65 KB) [file 228_2021_3138_MOESM1_ESM.docx]

**Supplementary**

**Legends supplementary information**

**Supplementary text:** The ADR-reporting assignment and pharmacovigilance teaching.

**Supplementary table 1:** E-questionnaire questions

**Supplementary table 2:** Knowledge/skills regarding the reporting of adverse drug reactions.

**Supplementary table 3:** Determinants influencing the adverse drug reaction (ADR) reporting rate in healthcare professionals.

**Supplementary** table 4: Results of evaluation of the pharmacovigilance reporting assignment.

**Supplementary figure 1:** ClinDoc scores of ADRs reported by SONs in the intervention group.

**Supplementary figure 2:** Percentages of student reflections on: “What to do when you suspect an Adverse Drug Reaction”.

**Supplementary figure 3:** Percentages of student reflections on: “What essential information is needed for a qualitative good report”.

**The ADR-reporting assignment and pharmacovigilance teaching**

All SONs agreed that the reporting assignment and group discussion were useful and that the assignment increased their alertness about medication and patient safety, and changed their practice in dealing with ADRs. Twelve SONs (20%) thought that the reporting assignment cost a lot of time, although it was more instructive than learning with fictive casuistry.

**Supplementary table 1:** E-questionnaire questions

| *Question* | | *Answer option* |
| --- | --- | --- |
| *General information only asked in the first e-questionnaire (control and intervention group)* | | |
| *1. Wat is your full name?* | | *Open question* |
| *2. Which college did you go to study the “prescribing qualification” course?* | | *“The Amstel Academy”, “Radboudumc Health Academy”, or “Wenckebach Instituut UMCG”* |
| *3. What is you sex* | | *Male or Female* |
| *4. What is your age category?* | | *< 35 years, 35-45 years, 45-55 years, >55 years* |
| *5. Which college did graduate from to be a “specialist oncology nurse”* | | *Open question* |
| *6. How many years of experience have you got as a nurse?* | | *Open question (only numbers)* |
| *7. Has the subject side effects been covered in any form during your training as (specialist) nurse?* | | *yes / no (if yes, please specify)* |
| *8. Has the subject ADR reporting been covered in any form during your training as (specialist) nurse?* | | *yes / no (if yes, please specify)* |
| *9. Have you reported an ADR to the Dutch pharmacovigilance centre Lareb before you enrolled in this module?* | | *yes / no (if yes, please specify)* |
| *General information only asked in the first e-questionnaire (only in the intervention group)* | | |
| *1. Can you indicate what you learned in this ADR-reporting assignment* | | *Open* |
| *2. Statements concerning the value of the reporting assignment*  *The ADR-reporting assignment…* | | *5 point Likert scale (1: strongly disagree – 5 strongly agree)* |
|  | *… was useful* |  |
|  | *… suited well within my practice* |  |
|  | *… improved attention for medication and patient safety* |  |
|  | *… is more instructive than learning with fictive casuistry* |  |
|  | *… cost a lot of time to do* |  |
|  | *… did change my practice in dealing with ADRs* |  |
| *3. Statements concerning the value of the plenary discussion of the reporting assignment*  *The ADR-reporting assignment discussion…* | | *5 point Likert scale (1: strongly disagree – 5 strongly agree)* |
|  | *… was useful* |  |
|  | *… is more instructive than learning with fictive casuistry* |  |
| *General information asked in all three e-questionnaires (control and intervention group)* | | |
| *1. What is your current work email address?* | | *Open question* |
| *2. In which hospitals (groups) are you currently working a specialist oncology nurse?*  *(if you are working at multiple hospitals, please mention all)* | | *Open question* |
| *3. At which departments are you working as a specialist oncology nurse?*  *(if you are working at multiple departments, please mention all)* | | *Open question* |
| *4. Are you doing outpatient clinic work independently?* | | *Yes / no* |
| *5. On average, how many prescriptions are you writing on a daily basis?* | | *0, 1-4, 5-9, 10-19, 20-29, >29* |
| *6. If you suspect a patient to have an adverse drug reaction, how would you act?* | | *Open question* |
| *7. I know where to report an ADR (in the Netherlands)?* | | *yes / no (if yes, please specify)* |
| *8. Have you reported an ADR to the Dutch pharmacovigilance centre Lareb before you enrolled in this module?* | | *yes / no (if yes, please specify)* |
| *9. How likely do you think the following outcomes will be if you report an ADR to the Dutch Pharmacovigilance Centre Lareb?* | | *7 point Likert scale (1: extremely unlikely – 7 extremely likely)* |
|  | *It contributes to the safe use of medicines.* |  |
|  | *Improves patient safety* |  |
|  | *Educates others about drug risks* |  |
|  | *Personally beneficial* |  |
|  | *Time consuming to report* |  |
|  | *Disrupts the normal workflow* |  |
|  | *Increases risk of malpractice* |  |
|  | *Breaks trust with patients* |  |
| *10. What is your opinion regarding the following statements?* | | *5 point Likert scale (1: extremely unlikely – 5 extremely likely)* |
|  | *Pharmacovigilance should be included as a core topic in the curriculum of all prescribers* |  |
|  | *Pharmacovigilance is well covered (up to now) in my curriculum* |  |
|  | *I do not know how I could report an ADR to the relevant authorities* |  |
|  | *Students can report ADRs during their clerk/internships* |  |
|  | *Reporting known ADRs makes no significant contribution to the reporting system.* |  |
|  | *With my present knowledge, I am very well prepared to report any ADRs in my future practice.* |  |
|  | *I believe that doctors are one of the most important healthcare professionals to report ADRs* |  |
|  | *I believe that pharmacists are one of the most important healthcare professionals to report ADRs* |  |
|  | *I believe that (specialist) nurses are one of the most important healthcare professionals to report ADRs* |  |
|  | *I believe serious and unexpected reactions that are not fatal or life-threatening during clinical trials should not be reported.* |  |
| *11. Could you indicate how likely it is you will report an ADR to the Dutch Pharmacovigilance Centre Lareb in the following situations:* | | *7 point Likert scale (1: extremely unlikely – 7 extremely likely)* |
|  | *I intend to report serious ADRs (ie. deaths, hospital admissions) that I will encounter to the competent authority.* |  |
|  | *I intend to report unknown ADRs that I will encounter to the competent authority.* |  |
|  | *I intend to report all ADRs that I will encounter to the competent authority.* |  |
| *12. I know which essential information is needed for a qualitative good ADR report* | | *yes / no (if yes, please specify)* |
| *13. What are the correct answers to the following statements?* | | *yes / no* |
|  | *All ADRs, irrespective of severity, must be reported.* |  |
|  | *Doctors should report serious ADRs even if uncertain that product caused the event.* |  |
|  | *Doctors should report serious ADRs even if do not have all details of event.* |  |
|  | *All serious ADRs are known before a drug is marketed.* |  |
|  | *Lareb does not disclose ADR reporter’s identity.* |  |
|  | *One can report ADRs anonymously to Lareb.* |  |
|  | *Adverse experiences with cosmetics and special nutritional products may be reported to Lareb* |  |
|  | *Adverse experiences with natural or homeopathic products may be reported to Lareb* |  |
|  | *Adverse experiences with vaccines may be reported to Lareb* |  |
|  | *One case reported by a doctor does not contribute much to knowledge about drug risks.* |  |
|  | *I have adequate knowledge of ADR reporting* |  |
|  | *Patients can report ADRs independent from a healthcare professional.* |  |

**Supplementary table 2:** Knowledge/skills regarding the reporting of adverse drug reactions.

|  | **Intervention group** | | | **Control group** | | | **Comparison** | | |
| --- | --- | --- | --- | --- | --- | --- | --- | --- | --- |
|  | T1 (n=65) | T2 (n=54) | T3 (n=47) | T1 (n=23) | T2 (n=19) | T3 (n=15) | T1 vs T1 | T2 vs T2 | T3 vs T3 |
| All ADRs, irrespective of severity, must be reported (*no) | 58.3 | 64.8 | 59.6 | 27.3 | 26.3 | 33.3 | **0.024** | **0.007** | 0.136 |
| Doctors should report serious ADRs even if uncertain that product caused the event (*yes) | 98.3 | 96.3 | 94.6 | 63.6 | 68.4 | 73.3 | **<0.001** | **0.003** | **0.047** |
| Doctors should report serious ADRs even if do not have all details of event (*yes) | 95.0 | 94.4 | 91.5 | 59.1 | 57.9 | 60.0 | **<0.001** | **0.001** | **0.009** |
| All serious ADRs are known before a drug is marketed (*no) | 88.3 | 85.2 | 83.0 | 50.0 | 47.4 | 26.7 | **0.001** | **0.002** | **<0.001** |
| Lareb does not disclose ADR reporter’s identity (*yes) | 88.3 | 85.2 | 72.3 | 63.6 | 57.9 | 73.3 | **0.021** | **0.023** | 1.000 |
| One can report ADRs anonymously to Lareb (*yes) | 78.3 | 77.8 | 76.6 | 54.5 | 57.9 | 46.7 | 0.051 | 0.135 | 0.051 |
| Adverse experiences with cosmetics and special nutritional products may be reported to Lareb (*yes) | 35.0 | 38.9 | 46.8 | 54.5 | 63.2 | 60.0 | 0.132 | 0.107 | 0.554 |
| Adverse experiences with natural or homeopathic products may be reported to Lareb (*yes) | 51.7 | 55.6 | 55.3 | 50.0 | 57.9 | 53.3 | 1.000 | 1.000 | 1.000 |
| Adverse experiences with vaccines may be reported to Lareb (*yes) | 96.7 | 92.6 | 93.6 | 72.7 | 63.2 | 66.7 | **0.004** | **0.005** | **0.016** |
| One case reported by a doctor does not contribute much to knowledge about drug risks (*no) | 75.0 | 90.7 | 91.5 | 40.9 | 47.4 | 33.3 | **0.008** | **<0.001** | **<0.001** |
| Patients can report ADRs independent from a healthcare professional (*yes) | 83.3 | 79.6 | 80.9 | 54.5 | 57.9 | 53.3 | **0.018** | 0.076 | **0.046** |

Supplementary table 2: Knowledge/skills regarding the reporting of adverse drug reactions (ADRs). Values are presented in percentage of correct answers (dichotomous-questions).

**Supplementary table 3:** Determinants influencing the adverse drug reaction (ADR) reporting rate in healthcare professionals.

| Determinants | Addressed in educational intervention |
| --- | --- |
| Attitudes relating to professional activity | |
| Financial incentives | Yes |
| Litigation concerns | Yes |
| Ambition to publish | Yes |
| Factors associated with ADR-related knowledge and attitudes | |
| Complacency  (only safe medications are marketed) | Yes |
| Insecurity  (determining whether or not a drug is responsible for a particular adverse reaction) | Yes |
| Diffidence  (fear of appearing ridiculous) | Yes |
| Indifference  (contributing to the general advancement of medical knowledge / lack of understanding of the purpose of reporting) | Yes |
| Ignorance  (only severe ADRs need to be reported) | Yes |
| Excuses made by professionals | |
| Lack of time | No |
| Different care priorities | No |
| Difficulty in accessing report form | Yes |
| Reporting process is extremely bureaucratic and complex | No |
| Aversion to disclosing confidential information | Yes |

**Supplementary table 3.** Determinants influencing the adverse drug reaction (ADR) reporting rate in healthcare professionals (by Lopez-gonzalez and Hazell (4-5)). Factors in green were addressed in the course and were positively affected, factors in red were NOT addressed in the course and were not affected.

**Supplementary** table 4: Results of evaluation of the pharmacovigilance reporting assignment.

|  | | | | |
| --- | --- | --- | --- | --- |
|  | | N | Median | IQR (25-75%) |
| The pharmacovigilance adverse drug reaction (ADR) reporting assignment …. | |  |  |  |
| ….. was useful. | | 60 | 5.0 | 4.0 – 5.0 |
| ….. fitted in with my work . | | 60 | 5.0 | 4.0 – 5.0 |
| ….. assignment improved attention for medication and patient safety. | | 60 | 5.0 | 4.0 – 5.0 |
| ….. was more instructive than learning with fictive casuistry. | | 60 | 4.0 | 4.0 – 5.0 |
| ….. cost a lot of time to do. | | 60 | 3.0 | 2.0 – 4.0 |
| ….. changed how I deal with ADRs. | | 60 | 4.0 | 4.0 – 5.0 |
|  |  | | | |
| The group discussion at the end of the reporting assignment …. | |  |  |  |
| ….. was useful. | | 60 | 5.0 | 4.0 – 5.0 |
| ….. assignment was more instructive than learning with fictive casuistry. | | 60 | 4.0 | 4.0 – 5.0 |

Supplementary table 2: Results of evaluation (on a 5-point Likert scale) of the pharmacovigilance reporting assignment.

**Supplementary figure 1:** ClinDoc scores of ADRs reported by SONs in the intervention group.

moderately

well

**Supplementary figure 1:** ClinDoc scores of ADRs reported by SONs in the intervention group.

**Supplementary figure 2:** Percentages of student reflections on: “What to do when you suspect an Adverse Drug Reaction”.

**Supplementary figure 2:** Percentages of student reflections on: “What to do when you suspect an Adverse Drug Reaction” categorised by ClinDoc tool groups. Darker bars represent students in the intervention group. Lighter coloured bars represent the control group.

**Supplementary figure 3:** Percentages of student reflections on: “What essential information is needed for a qualitative good report”.

**Supplementary figure 3:** Percentages of student reflections on: “What essential information is needed for a qualitative good report” categorised by ClinDoc tool groups. Darker bars represent students in the intervention group. Lighter coloured bars represent the control group.
